# Effects of drying and storage conditions on the stability of TSH in blood spots

**DOI:** 10.20945/2359-3997000000026

**Published:** 2018-03-23

**Authors:** Patrícia Künzle Ribeiro Magalhães, Carlos Henrique Miranda, Fernando Crivelenti Vilar, André Schmidt, Roberta Rodrigues Bittar, Giselle Aparecida Caixe de Carvalho Paixão, Edson Zangiacomi Martinez, Léa Maria Zanini Maciel

**Affiliations:** 1 Universidade de São Paulo Universidade de São Paulo Departamento de Medicina Interna, Faculdade de Medicina de Ribeirão Preto Divisão de Endocrinologia Ribeirão Preto SP Brasil Divisão de Endocrinologia, Departamento de Medicina Interna, Faculdade de Medicina de Ribeirão Preto, Universidade de São Paulo (FMRP-USP), Ribeirão Preto, SP, Brasil; 2 Universidade de São Paulo Universidade de São Paulo Faculdade de Medicina de Ribeirão Preto Departamento de Medicina Social Ribeirão Preto SP Brasil Departamento de Medicina Social, Faculdade de Medicina de Ribeirão Preto, Universidade de São Paulo (FMRP-USP), Ribeirão Preto, SP, Brasil

**Keywords:** Neonatal screening, TSH, congenital hypothyroidism, filter paper storage

## Abstract

**Objective:**

To evaluate the influence of sample drying and storage temperature on TSH stability in neonatal screening.

**Subjects and methods:**

Blood samples from 29 adult volunteers as a surrogate for neonatal blood (10 with normal TSH, 9 with overt hypothyroid and 10 with subclinical hypothyroidism) were spotted on filter paper and dried at 22°C or 35°C for 3 hours. The samples were then stored at 22°C, -4°C, or -20°C, and TSH measurements were performed at day 0 (D0), D7, D30, D60, D180, and D360 of storage.

**Results:**

The drying temperature did not interfere with TSH measurement on D0. TSH values remained stable up to D30 when stored at 22°C and were stable up to D60 when stored in a refrigerator or freezer. Samples stored at 22°C had a greater decrease in TSH values than samples stored in a refrigerator or a freezer.

**Conclusions:**

Freezer storage is not advantageous compared to storage in the refrigerator. At the end of one year, if confirmation of the initial result is required, a reduction of TSH concentrations should be taken into account.

## INTRODUCTION

Neonatal screening for congenital hypothyroidism (CH) in order to prevent neurological deficit is accomplished through measurement of thyroid stimulating hormone (TSH) in dried blood spots on filter paper (1).

Despite the simplicity of this method, which does not require venipuncture and simplifies sample transportation from distant regions to a central laboratory, questions arise about the possible interference of sample drying, storage and transportation under different physical conditions and of the quality of filter paper with the stability of the hormones (2–8).

This study was conducted to evaluate the influence of sample drying and storage temperature on TSH stability along 1 year.

## SUBJECTS AND METHODS

### Sample collection, drying and storage

This study was conducted at the University Hospital, Ribeirão Preto Medical School, USP (HCFMRP-USP), one of four newborn screening reference centers in the State of São Paulo, Brazil.

Adult blood was tested as a surrogate for neonatal blood. Two venous blood samples (4 mL in a sodium heparin tube and 4 mL in a clotting activator tube [BD Vacutainer^®^ tubes; catalog numbers 367835 and 368774, respectively]) were collected from 29 adult volunteers aged 21-76 years: 10 had normal serum TSH (TSH = 0.4 – 4 µIU/mL), 9 had a serum TSH of ≥ 20 µIU/mL, and 10 had a serum TSH of > 4.0-20 µIU/mL during routine tests performed at the Newborn Screening and Thyroid Laboratory of HCFMRP-USP.

Six filter paper discs (Whatman™ 903 Neonatal Screening Cards, Life Sciences, GE Healthcare, US) per subject were spotted with 50 µL of heparinized blood. Three discs from each individual were dried horizontally at room temperature (RT, 22°C) and 50% air relative humidity (ARH) for 3 hours. The remaining three discs were dried horizontally in an oven at 35°C and 65% ARH for 3 hours, simulating the temperature in tropical countries like ours. After drying, the samples were wrapped in aluminum foil envelopes and stored under three different conditions (1 disc per patient in each condition): at RT (22°C), in a refrigerator (4°C), or in a freezer (-20°C).

### Laboratory determinations

Serum TSH was measured by a chemiluminescent assay (Immulite 2000, Diagnostic Products Corporation, Los Angeles, CA), with an interassay error of < 10%. The total blood TSH equivalent was calculated from the serum TSH value according to the patient's hematocrit.

TSH measurements of the filter paper blood spots under all conditions were performed by the AutoDELFIA^®^ Neonatal hTSH Kit (PerkinElmer, Wallac Oy, Finland) at six different times: the same day of sample collection (day 0 [D0]), D7, D30, D60, D180, and D360 of storage. The intra-assay and interassay coefficients of variation were < 10%.

### Statistical analysis

Serum TSH, hematocrit, total blood TSH equivalent and baseline values of TSH in blood collected on filter paper were expressed in median and range.

TSH values in blood collected on filter paper were log transformed due to their non-Gaussian distribution. The transformed data were analyzed by using mixed effects linear models (9), considering the effects of days of storage (D0, D7, D30, D60, D180 and D360), storage temperatures (RT, 4°C, and -20°C), and interactions between days of storage and storage temperatures.

Analyses were performed in SAS 9.3 (10), with the level of significance set at 0.05.

The study was approved by the Research Ethics Committee of HCFMRP-USP (protocol No. 13094/2011).

## RESULTS

Median serum TSH for all samples was 9.7 μUI/mL (range: 0.5-301) and the hematocrit was 38.7% (range: 24-45). The median total blood TSH equivalent was 6.0 μUI/mL (range: 0.3-172.3) and confirmed the initial dried blood TSH concentrations. The median baseline values of TSH in blood collected on filter paper were 6.0 µIU/mL whole blood (WB) (range: 0.4-154) for samples dried at RT and 6.0 µIU/mL WB (range: 0.4-140) for samples dried in an oven. After log transformation, the geometric mean and confidence interval of the TSH values at D0 were 6.4 (3.4-12.0) and 6.0 (3.2-11.3) µIU/mL WB for the samples dried at RT and for the samples dried in an oven, respectively, with no significant difference between them (*P* = 0.06).

**Figure 1:**
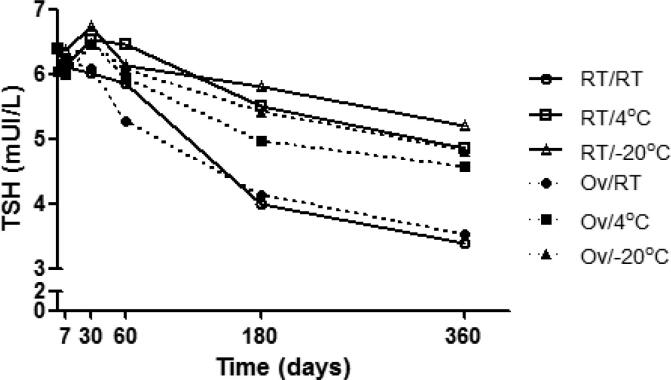
Variation of geometric mean TSH concentration (µIU/mL) along time under the different drying and storage conditions. RT/RT = samples dried and stored at room temperature; RT/4°C = samples dried at room temperature and stored in a refrigerator; RT/-20°C = samples dried at room temperature and stored in a freezer; Ov/RT = samples dried in an oven and stored at room temperature; Ov/4°C = samples dried in an oven and stored in a refrigerator; Ov/-20°C = samples dried in an oven and stored in a freezer.

TSH values over time under different storage conditions demonstrated the same behavior in both the samples dried at RT and those dried in an oven, *i.e.*, they were stable up to D30 when stored at RT and were stable up to D60 when stored in a refrigerator or a freezer (Table 1 and Figure 1).

**Table 1:** Thyroid stimulating hormone (TSH) concentrations (µIU/mL whole blood) in blood samples collected on filter paper and stored under different temperatures (room temperature [RT], 4°C, and -20°C) for 1 year

	Dried at RT				Dried in an Oven			
Days	RT	4°C	-20°C	P values	RT	4°C	-20°C	P values
D0	6.4				6.0			
	(3.4, 12.0)				(3.2, 11.3)			
D7	6.1	6.1	6.4	RT vs 4°C P = 0.86	6.2	6.0	6.2	RT vs 4 °C P = 0.45
	(3.3, 11.4)	(3.3, 11.5)	(3.4, 12.0)	RT vs -20°C P = 0.33	(3.3, 11.6)	(3.2, 11.2)	(3.3, 11.6)	RT vs -20 °C P = 0.98
	P = 0.27	P = 0.35	P = 0.89	4°C vs -20°C P = 0.42	P = 0.59	P = 0.83	P = 0.57	4oC vs -20 °C P = 0.44
D30	6.0	6.5	6.7	RT vs 4°C P = 0.06	6.1	6.5	6.5	RT vs 4 °C P = 0.20
	(3.2, 11.3)	(3.5, 12.2)	(3.6, 12.6)	RT vs -20°C P < 0.01	(3.3, 11.4)	(3.5, 12.1)	(3.5, 12.1)	RT vs -20 °C P = 0.17
	P = 0.16	P = 0.63	P = 0.20	4°C vs -20°C P = 0.41	P = 0.86	P = 0.14	P = 0.12	4oC vs -20 °C P = 0.95
D60	5.9	6.4	6.1	RT vs 4°C P = 0.02	5.3	6.0	6.1	RT vs 4 °C P = 0.01
	(3.1, 11.0)	(3.4, 12.1)	(3.3, 11.5)	RT vs -20°C P = 0.25	(2.8, 9.9)	(3.2, 11.1)	(3.3, 11.4)	RT vs -20 °C P <0.01
	P = 0.04	P = 0.83	P = 0.34	4°C vs -20°C P = 0.24	P < 0.01	P = 0.73	P = 0.91	4oC vs -20 °C P = 0.65
D180	4.0	5.5	5.8	RT vs 4°C P < 0.01	4.1	5.0	5.4	RT vs 4 °C P <0.01
	(2.1, 7.5)	(2.9, 10.3)	(3.1, 10.9)	RT vs -20°C P < 0.01	(2.2, 7.8)	(2.7, 9.3)	(2.9, 10.1)	RT vs -20 °C P <0.01
	P < 0.01	P < 0.01	P = 0.02	4°C vs -20°C P = 0.21	P < 0.01	P <0.01	P = 0.02	4oC vs -20 °C P = 0.06
D360	3.4	4.9	5.2	RT vs 4°C P < 0.01	3.5	4.6	4.8	RT vs 4 °C P <0.01
	(1.8, 6.4)	(2.6, 9.1)	(2.8, 9.7)	RT vs -20°C P < 0.01	(1.9, 6.6)	(2.5, 8.6)	(2.6, 9.0)	RT vs -20 °C P <0.01
	P < 0.01	P < 0.01	P < 0.01	4°C vs -20°C P = 0.10	P < 0.01	P <0.01	P <0.01	4oC vs -20 °C P = 0.27

TSH values are reported as geometric means with their respective confidence intervals (95%CI). *P* values below the geometric mean refer to the comparison with the D0 mean. D0, TSH measurement at the same day of sample collection; D7, D30, D60, D180 and D360, TSH measurement after 7, 30, 60, 180, and 360 days of storage, respectively.

At the end of 360 days, the median reduction of TSH concentrations compared to baseline values when the filter paper was dried at RT and stored at RT, in a refrigerator, or in a freezer, was 47.7%, 16.8%, and 10.1%, respectively. When the paper was dried in an oven, the mean reduction of TSH concentration was 37.4%, 12.8% and 9.2% when the filter paper was stored at RT, in a refrigerator or in a freezer, respectively. Regardless of the drying conditions, a greater decrease of TSH values occurred in samples stored at RT than in samples stored in a refrigerator or a freezer (*P* < 0.001), with no significant difference between storage in a refrigerator or a freezer (Table 1 and Figure 1).

## DISCUSSION

The use of blood obtained by heel puncture and dried on filter paper has greatly facilitated newborn screening. Although the pertinent literature is quite limited (2–8), it is undeniably important to establish the best drying and storage conditions of blood samples collected on filter paper for newborn screening. The samples are frequently exposed to different climate conditions during drying and transport to the laboratories, a fact that may influence the test results. These factors are extremely relevant for the National Newborn Screening Program in view of the climatic conditions of some Brazilian regions and those of other tropical countries, with high temperatures and humidity, and considering that the test cards are transported in ambulances, buses, or postal transport cars, in most cases without proper packaging. Additionally, in Brazil, the cards with filter paper are usually stored in a refrigerator until the screening tests are performed. After the results are obtained, the cards are stored at RT so the samples can be reutilized in the future for confirmation of the initial results in case of diagnostic doubts.

The current study demonstrated that drying temperatures of up to 35°C for 3 hours do not interfere with the basal TSH results. Sample storage conditions, however, interfered with the TSH values observed. Regardless of the drying conditions (RT or oven), the samples remained stable for a longer period of time (up to D60) when stored in a refrigerator or freezer than when stored at RT (up to D30), with no difference between storage in a refrigerator or a freezer.

The present data disagree with those reported by Chen and cols. (5) who observed that the TSH values in blood collected by newborn heel puncture and dried on filter paper were higher when the samples were dried in room air than under direct sunlight (40-60°C) or in a heater (60-80°C). However, the drying temperatures used by Chen and cols. (5) were higher than those used in the current study (35°C), which are close to those naturally occurring in Brazil during the summer. In addition, some of the samples in Chen and cols. (5) were directly exposed to sunlight, a fact that may have contributed to the reduction of TSH. Within this context we may refer to the work of Davis & Poholek (2) who demonstrated that direct irradiation with sunlight or exposure to a high temperature (60-64°C), especially in combination with exposure to higher humidity, may reduce blood extraction from the filter paper, reduce thyroxine (T_4_) values, and may consequently yield false-negative results within a few days after collection. It should be noted, however, that T_4_ is more susceptible than TSH to the effects of improper sample drying and storage, representing an additional factor favoring TSH determination as the method of choice for newborn CH screening (4,8).

Confirming the stability of TSH up to D30 regardless of drying or storage conditions, Waite and cols. (4) did not detect differences in TSH results during 1 month of storage of blood samples collected on filter paper, dried at room temperature for several hours, and stored under different temperature and humidity conditions. The stability of TSH in this study (30 days at RT and up to 60 days in the refrigerator or freezer) was shorter than the 36 months reported by Lando and cols. (6) for samples collected from newborns on filter paper and stored in a refrigerator (2-8°C), and also shorter than the 2.7, 4.1 and 6.5 years for samples stored at RT, 4°C and -20°C, respectively, reported by El Ezzi and cols. (7). However, the determinations performed by Lando and cols. (6) over a period of 60 months were not carried out on samples from the same individual, a fact that limits this comparison. El Ezzi and cols. (7) used a sealed plastic bag containing silica gel for samples storage, which may have increased sample stability. This interpretation is supported by Adam and cols., (8) who found that TSH and T_4_ degradation on filter paper is three and ten times greater, respectively, in samples stored at higher humidity. In addition, other factors such as the quality of the filter paper used, the way the blood was placed on filter paper, the time of blood elution, and even the method of hormone determination may interfere with TSH results and stability (6).

It must be mentioned, that our study has an important limitation: the great variability of the TSH values of the samples. In Brazil, the cut-off level of neonatal TSH used by the newborn screening reference centers is around 6-10 µIU/mL WB. Imprecision and a moderate degree of instability will not affect the screening decision for a TSH level of < 4 mIU/L blood WB (normal) or > 20 mIU/L blood WB, but could lead to failure diagnosis of affected children who had neonatal TSH close to cut-off levels. However, in this study, an analysis using only the samples with serum TSH of 4.5–20 µIU/mL would have a low accuracy because of the small number of samples (N = 10).

In conclusion, although adult samples were used instead of newborn blood, the results of the current study contribute to better quality control of TSH results obtained in newborn screening tests and indicate the lack of influence of high temperatures for sample drying. In addition, these results suggest that, under ideal conditions, the sample should be placed in a refrigerator for long-term storage, something that does not routinely occur in Brazilian Newborn Screening laboratories. Even so, if confirmation of the initial result should become necessary after 6 months of storage, it is necessary to consider the possibility of reduced TSH concentrations due to loss of hormone stability inherent to storage, mainly in samples close to cut-offs levels.
